# Effect of Temperature on Polyamine Oxidase Genes in *Skeletonema dohrnii*

**DOI:** 10.3390/ijms26031048

**Published:** 2025-01-26

**Authors:** Wei Teng, Jun Sun

**Affiliations:** 1 Research Centre for Indian Ocean Ecosystem, Tianjin University of Science and Technology, Tianjin 300457, China; tengwei990412@163.com; 2 Institute for Advanced Marine Research, China University of Geosciences, Guangzhou 511462, China

**Keywords:** abiotic stress, diatom, polyamine metabolism

## Abstract

In our experiments, we investigated the effect of temperature on diatom polyamine metabolism using *Skeletonema dohrnii* as an experimental algal species. We set three different temperature conditions for incubation and selected *Skeletonema dohrnii* in the exponential growth period, and analyzed basic physiological parameters, polyamine composition and content, and polyamine oxidase (PAO) gene expression at different temperatures. The results showed that low temperatures led to a decrease in growth rate, an increase in biogenic silica content, an increase in the content of putrescine and spermine, a decrease in the concentration of spermidine, and a down-regulation of PAO gene expression. In addition, high temperature led to an increase in growth rate, a significant change in the concentration of putrescine and spermine, and an increase in spermidine. These findings suggest that changes in temperature affect the growth rate of algae, low temperature increases the biogenic silica content of diatoms, different temperature stresses lead to different kinds of polyamine changes in diatoms, and the PAO gene may play a role in regulating the response of algae to temperature changes. This study lays a foundation for further exploration of the function of the PAO gene in *Skeletonema dohrnii*.

## 1. Introduction

According to the latest survey report, global temperatures will rise between 1 °C and 5 °C [1]. In this case, biomes around the world will be seriously affected [2,3,4]. Phytoplankton are thermophiles and cannot regulate their own temperature. Therefore, they are extremely sensitive to temperature, and changes in temperature have a great impact on them, and temperature controls cell metabolism and stability by regulating the activity of certain enzymes [5,6,7]. Phytoplankton play a very important role in the ecological environment, which is the basis of the ecosystem food chain and the main source of primary productivity [8,9]. Therefore, studying the effects of temperature on phytoplankton physiology and cellular metabolism is crucial for better predicting the evolution of ecosystems in changing environments and is likewise immensely helpful in exploring the mechanisms by which phytoplankton respond to temperature.

Diatoms are unicellular photosynthetic eukaryotes that are recognized as being of global significance in biogeochemical cycles and the functioning of aquatic food webs [10,11]. They constitute a significant portion of the aquatic biomass, especially during pronounced seasonal phytoplankton blooms, and are estimated to account for 20% of the Earth’s total primary plant production [8,12]. Due to their complex evolutionary history [13], diatoms have a “mixed-match genome” [14], which equips them with a variety of potentially advantageous traits, such as rigid silicified cell walls, the ability to rapidly respond to changes in ambient light, the formation of a stationary phase, and engagement in urea cycling [15]. Typically, planktonic diatoms are well suited to environments with intermittent light and nutrient availability. However, they are especially abundant in nutrient-rich regions, such as the polar zones, upwelling areas, and coastal environments [16], highlighting their remarkable adaptability to diverse ecological niches and biomes.

*Skeletonema dohrnii* was selected in this study as an important factor contributing to the occurrence of red tide [17]. It has been noted that in areas where red tides are frequent, there are periodic variations between diatom red tides and methanotrophic red tides due to environmental factors and ecological processes [18]. Studies of red tides have shown that environmental conditions are key in determining which species become dominant during red tide outbreaks, with temperature being a key factor in promoting diatom growth. In addition, during red tide outbreaks, the concentration of polyamines in seawater tends to increase with phytoplankton blooms [19,20].

**Figure 1:**
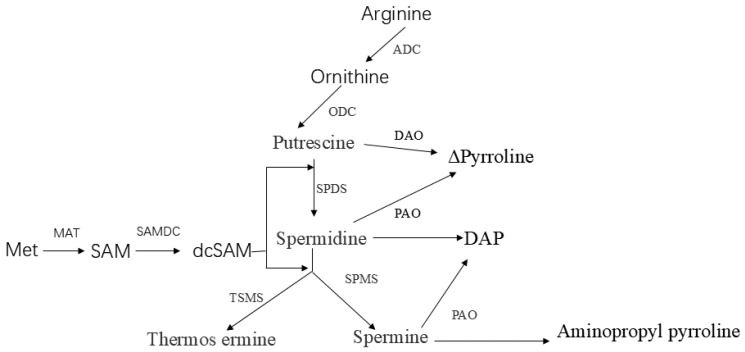
A simple pathway for polyamine anabolism in microalgae (adapted from Miguel A. Blázquez [29]).

Polyamines, which are organic polymeric cations commonly present in most organisms at physiological pH, serve as crucial growth regulators during the growth processes of phytoplankton [21,22,23]. In microalgae, polyamines are associated with both cell growth and the accumulation of metabolites [24,25,26]. Among the polyamines, the most extensively studied are putrescine (PUT), spermidine (SPD), and spermine (SPM), which are widely distributed and relatively abundant. In addition to these, less common polyamines such as diaminopropane (DAP), norspermidine (NSPD), and norspermine (NSPM) have recently garnered attention for their potential involvement in newly identified biochemical pathways. The dominant polyamine species differ across algae, potentially due to variations in their evolutionary histories [27,28]. The metabolic pathways of polyamines in microalgae remain poorly understood compared to those studied in higher plants. Nevertheless, higher plant polyamine metabolism can serve as a reference for modeling these pathways in microalgae (Figure 1). Interestingly, the chemical synthesis of polyamines in microalgae diverges from that of higher plants, with a key distinction lying in the starting point: polyamine synthesis in microalgae typically begins with arginine. This precursor undergoes decarboxylation and enzymatic reactions to form putrescine, which is further converted into H_2_O_2_, 4-aminobutanal, and, ultimately, 1-pyrroline through the catalytic action of diamine oxidase (DAO). Moreover, putrescine serves as a precursor for the synthesis of triamines and tetraamines via the activity of aminopropyl transferases. Specifically, spermidine synthase (SPDS) transfers an aminopropyl group from decarboxylated S-adenosyl methionine (dcSAM) to putrescine, producing SPD, which can then be converted into SPM through the addition of another aminopropyl group by spermine synthase (SPMS). Alternatively, TSPM synthase (TSPMS) can attach the aminopropyl group to the opposite end of the SPD molecule to produce thermospermine (TSPM), an isomer of SPM. A crucial step in these processes is the generation of dcSAM by SAM decarboxylase (SAMDC), which provides the aminopropyl donor for the reactions catalyzed by aminopropyl transferases.

Polyamines are widely found in animals, plants and bacteria. Current research on the role of polyamines has focused on plants and animals. Polyamines play an important role in plant growth, development and other physiological processes. Polyamines play a role in mitigating abiotic stress in plants [30,31,32]. Similar effects have been found in polyamine studies in marine microalgae. Previously, it was found that silicate concentration leads to changes in polyamine concentration and gene expression of polyamine oxidase in diatoms [33]. In general, there are few studies on the response of microalgal polyamines to environmental stresses, and the molecular regulatory mechanisms of polyamine metabolism are also rarely investigated.

Polyamine oxidases (PAOs) are FAD-dependent enzymes involved in the catabolism of polyamines [34]. They catalyze the oxidation of spermidine (Spd), spermine (Spm), and/or their acetylated derivatives at secondary amino groups [35]. The roles of plant PAOs in polyamine catabolism can be grouped into two primary categories. The first category includes PAOs that mediate the terminal catabolism of polyamines. These enzymes catalyze reactions that oxidize Spd or Spm, producing hydrogen peroxide, 1,3-diaminopropane (DAP), and 4-aminobutane (in the case of Spd oxidation) or N-(3-aminopropyl)-4-aminobutane (in the case of Spm oxidation). The second category includes PAOs that carry out the trans-conversion of polyamines, such as the conversion of Spm to Spd and Spd to putrescine (Put). These reactions also generate hydrogen peroxide as a by-product of their catalytic activity. The terminal catabolic pathway of polyamines is specifically activated in the extracellular compartment, whereas the trans-conversion pathway occurs within the intracellular cytoplasmic space, predominantly in the peroxisomes [36].

The purpose of this study is to analyze how *Skeletonema dohrnii* cells respond to different temperatures. At the same time, *Skeletonema dohrnii*, as a red tide algal species, can also be used to investigate the effect of temperature on red tide. This study will explore factors such as cell growth rate, Fv/Fm, chlorophyll content, biosilicon content and polyamine composition as well as content. In addition, we will evaluate the expression of polyamine oxidase genes.

## 2. Results

### 2.1. Physiological Property

**Figure 2:**
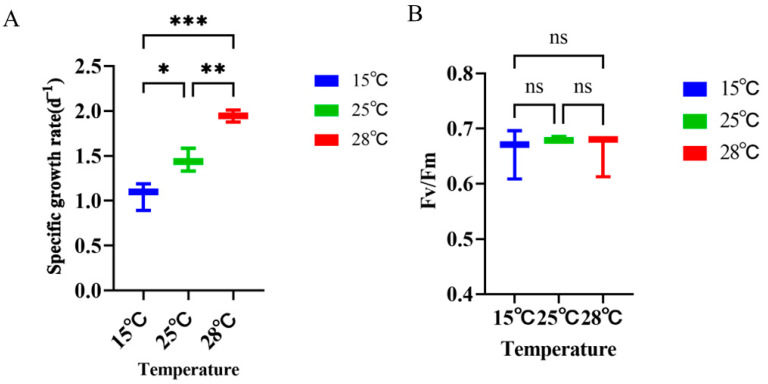
Physiological traits of *Skeletonema dohrnii* cultured under different temperature conditions. (**A**): specific growth rate of *Skeletonema dohrnii* at different temperatures; (**B**): Fv/Fm of *Skeletonema dohrnii* at different temperatures; (ns indicates no significance; * indicates *p* < 0.05; ** indicates *p* < 0.01; *** indicates *p* < 0.001).

**Figure 3:**
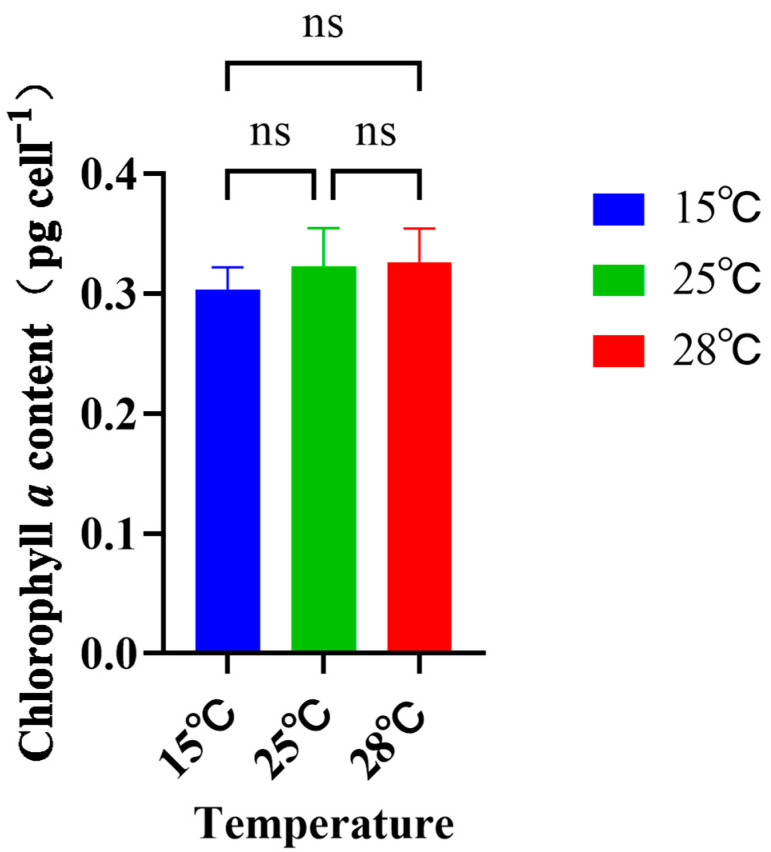
Chlorophyll *a* content of *Skeletonema dohrnii* cultured under different temperature conditions (ns indicates not significant).

The change in temperature significantly affected the growth of *Skeletonema dohrnii* (Figure 2A). The growth rate of *Skeletonema dohrnii* was significantly lower at 15 °C than at the normal temperature of 25 °C. In particular, the maximum growth rate of 1.58 times d^−1^ at 25 °C was significantly higher than the rate of 1.18 times d^−1^ at 15 °C. At 28 °C, the growth rate of *Skeletonema dohrnii* was significantly higher than the rate at 25 °C. The maximum growth rate of 2.01 times d^−1^ at 28 °C was higher than the maximum growth rate at 25 °C. From Figure 1, we can see that the growth rate at 15 °C was significantly lower than the rate at 25 °C, and the growth rate at 15 °C was significantly lower than the rate at 28 °C. Similarly, the growth rate at 25 °C was significantly lower than that at 28 °C, so it can be seen that the increase in temperature promoted the growth rate of *Skeletonema dohrnii*. In addition, all Fv/Fm values ranged from 0.61–0.70 with no significant difference among the three treatments, indicating that temperature did not significantly affect the photosynthetic performance of the algae during the experiment (Figure 2B). The content of chlorophyll *a* fluctuated with time in *Skeletonema dohrnii* under each temperature condition (Figure 3). However, the chlorophyll concentrations of *Skeletonema dohrnii* under the three temperature incubations showed no significant differences.

### 2.2. Biosilicon Content

**Figure 4:**
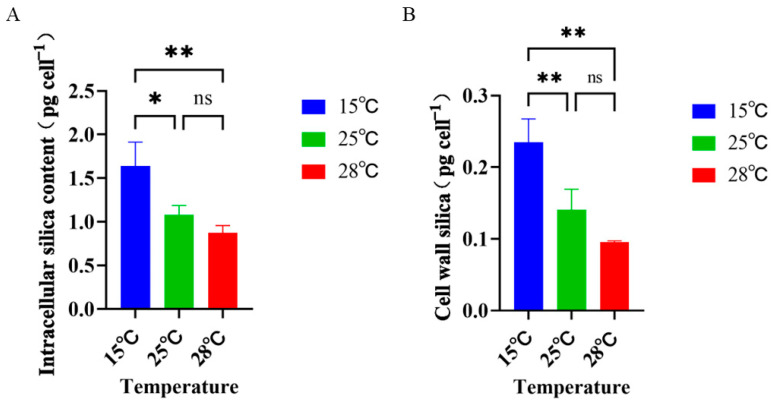
Biosilica content of *Skeletonema dohrnii* cultured under different temperature conditions. (**A**): Intracellular silica content of *Skeletonema dohrnii* at different temperatures; (**B**): Cell wall silica content of *Skeletonema dohrnii* at different temperatures (ns indicates non-significant; * indicates *p* < 0.05; ** indicates *p* < 0.01).

The biogenic silica content (pg cell^−1^) of *Skeletonema dohrnii* cultured at different temperature culture conditions was different (Figure 4). In terms of intracellular silicon content (Figure 4A), the biosilicon content was 1.078 ± 0.082 at the 25 °C (normal) culture condition, while the biosilicon content at 15 °C (low temperature) was 1.51 times higher than that of the 25 °C culture condition, reaching 1.638 ± 0.291. The biosilicon content at 28 °C (high temperature) was 0.81 times higher than that of the 25 °C culture condition, which reached 0.876 ± 0.074. Changes in cell wall silica content were consistent with intracellular silica content (Figure 4B). The cell wall silica content at 25 °C (normal) incubation condition was 0.14 ± 0.033, whereas the biogenic silica content under the incubation condition at 15 °C (low temperature) was 1.67 times higher than that of the incubation condition at 25 °C, which reached 0.234 ± 0.029. The biogenic silica content under the incubation condition at 28 °C (high temperature) was 1.67 times higher than that of the incubation condition at 28 °C (high temperature). The biosilica content was 0.67 times higher than that of the 25 °C incubation condition, reaching 0.095 ± 0.002. However, it was determined that the change from normal in the high-temperature group was not significant.

### 2.3. Polyamine Concentration

**Figure 5:**
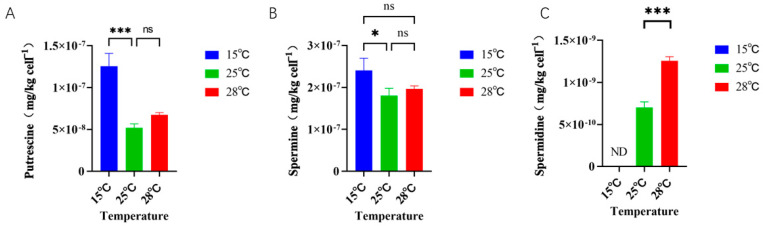
Concentration of polyamines in *Skeletonema dohrnii* cultured under different temperature conditions. (**A**): putrescine content of *Skeletonema dohrnii* at different temperatures; (**B**): spermine content of *Skeletonema dohrnii* at different temperatures; (**C**): spermidine content of *Skeletonema dohrnii* at different temperatures (ND indicates none detected; ns indicates non-significance; * indicates *p* < 0.05; *** indicates *p* < 0.001).

The concentration of polyamines differed in different temperature conditions of culture (Figure 5). There was a significant difference in the concentration of putrescine in *Skeletonema dohrnii* cultured at 15 °C (low temperature) compared to 25 °C (normal temperature), with the putrescine concentration in the low-temperature group being 2.53 times higher than that in the normal group. In contrast, there was no significant difference in the concentration of putrescine in *Skeletonema dohrnii* under the 28 °C (high temperature) culture condition (Figure 5A). The distribution of spermine concentration in different treatment groups was similar to that of putrescine (Figure 5B). There was a significant difference between the spermine concentration of *Skeletonema dohrnii* under low-temperature culture conditions and that of the normal-temperature group, with the spermidine concentration in the low-temperature group being 1.39 times higher than that of the normal group, whereas there was no significant difference between the high-temperature group and that of the normal-temperature group. The distribution of spermine content under different temperature conditions was different from both putrescine and spermidine (Figure 5C). Spermidine was not detected in the 15 °C culture condition, and there was also a significant difference in the content of spermidine between the 25 °C and 28 °C incubation conditions. The spermidine content in the 28 °C culture condition was 1.78 times higher than that in the 25 °C group.

### 2.4. Polyamine Oxidase Gene Expression

**Figure 6:**
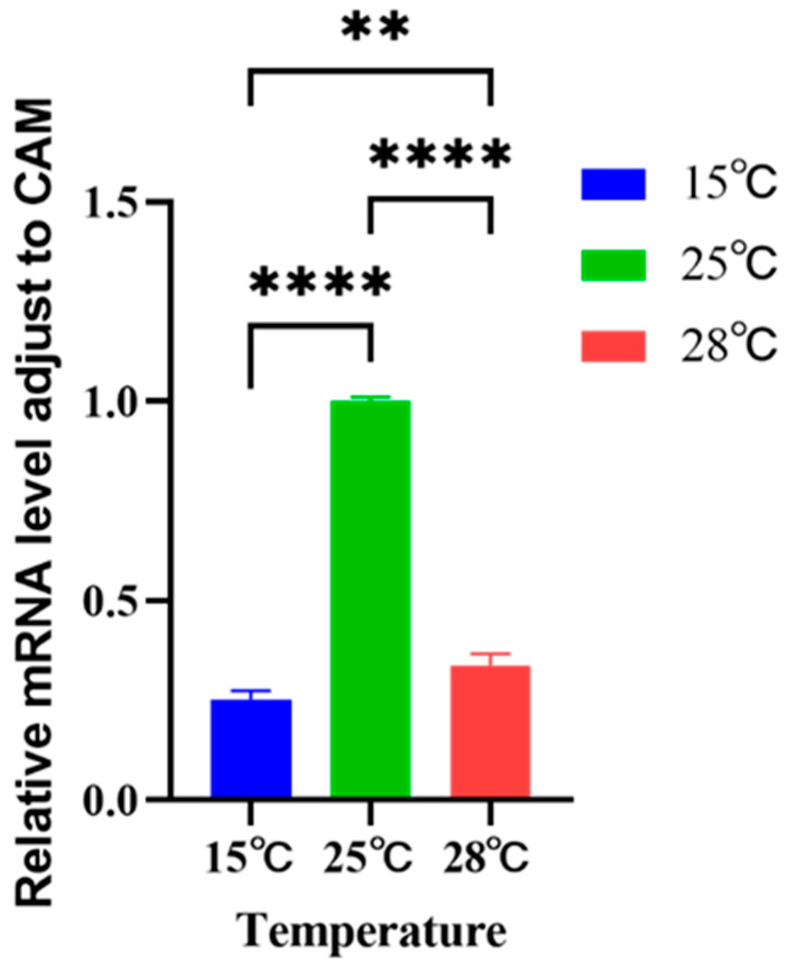
Expression of polyamine oxidase gene in *Skeletonema dohrnii* cultured at different temperatures (** indicates *p* < 0.01; **** indicates *p* < 0.0001).

Polyamine oxidase is an enzyme involved in the regulation of polyamine levels in vivo, and its expression was significantly different under different temperature conditions (Figure 6). The polyamine oxidase protein gene expression of *Skeletonema dohrnii* cultured at a low temperature of 15 °C was lower than that of *Skeletonema dohrnii* cultured at a normal temperature of 25 °C, and the polyamine oxidase protein gene expression of *Skeletonema dohrnii* cultured at a low temperature of 15 °C was 0.25 times higher than that of A. *Skeletonema dohrnii* cultured at a normal temperature of 25 °C. The polyamine oxidase protein gene expression of *Skeletonema dohrnii* cultured at a high temperature of 28 °C was lower than that of *Skeletonema dohrnii* cultured at a normal temperature of 25 °C, and the polyamine oxidase protein gene expression of *Skeletonema dohrnii* cultured at a high temperature of 28 °C was 0.3 times of that of *Skeletonema dohrnii* cultured at a normal temperature of 25 °C.

## 3. Discussion

In recent years, research on the effects of ocean warming on phytoplankton has always been devoted to the study of the impacts of ocean warming on phytoplankton, and most of the studies have focused on the impacts on the dynamics of phytoplankton communities. Few studies have focused on the molecular mechanisms behind the phytoplankton response to ocean warming and the changes in differential gene expression. In this study, we investigated the response of an ecologically significant diatom in polyamine metabolism at different temperatures by combining the changes in specific gene expression and physiological traits and found differences in the expression of key genes of polyamine metabolism at different temperatures, so as to provide a reference for the study of molecular regulatory mechanisms in the response of marine diatoms to temperature stress.

### 3.1. Response of Physiological Traits to Temperature in Skeletonema dohrnii

Previous studies have shown that warmer temperatures have a positive or neutral effect on the growth of marine phytoplankton [37,38,39,40]. For example, cyanobacteria, when cultured individually in batches, have higher growth rates at higher temperatures than at normal temperatures [38]. It has also been shown that functional types of phytoplankton may have different temperature coefficients (Q10), maximum-temperature dependence of growth, and temperature ranges, which would lead to different responses to different degrees of temperature change. In response to global modeling of future temperatures, these differences lead to taxon-specific predictions of growth and geographic distribution, with low-latitude coccolithophores facing considerable reductions and cyanobacterial growth rates increasing substantially. A single effect of temperature change may alter the global community structure of phytoplankton due to significant differences in thermal response among functional phytoplankton types [41]. In the present study, it was observed that the growth rate of *Skeletonema dohrnii* algae incubated under different temperature conditions was directly proportional to the increase in temperature, which increased with the increase in temperature.

The Fv/Fm parameter is generally used to assess whether the cells are stressed by environmental stress, and the value of Fv/Fm is generally constant in the absence of stress from environmental stressors. In the present study, the change in temperature did not significantly change the Fv/Fm values of *Skeletonema dohrnii* throughout the experiment, indicating that the cells remained relatively active throughout the experiment. Similarly, the Fv/Fm values of *Skeletonema dohrnii* did not change significantly after 400 generations of selection in a high-temperature environment [42]. In conclusion, *Skeletonema dohrnii* under experimental temperature stress still maintains constant PSII functional efficiency. It is noteworthy that there was no significant change in the chlorophyll *a* content within the experimental temperature. Similarly, some species showed no significant change in chlorophyll content in response to changes in temperature. For example, the chlorophyll content remained unchanged in *Chaetoceros* sp., *Rhodomonas* sp., *Cryptomonas* sp., and *Isochrysis* sp. grown at 25 °C, 27 °C, 30 °C, 33 °C, and 35 °C [43].

Notably, we found that the biogenic silica content was higher in the low-temperature than in the normal- and high-temperature groups in the experimental temperatures. Some researchers have found that diatom cell size decreases with increasing temperature [44]. The size of the cell volume affects the biogenic silica content, which is similar to the results of the present study. Physically, diatoms with higher densities at lower temperatures appear to be better able to contribute to the “ballasting effect” (referring to the ability of BSi, as the major silicate biomineral, to largely promote POC transport from the surface to deeper ocean), whereas diatom blooms accelerate at higher temperatures and have lower silica content, which may be detrimental to carbon sequestration in the oceans [45].

### 3.2. Response of Polyamine Content to Temperature

Temperature is widely acknowledged as a critical environmental factor influencing algal growth. Polyamines (PAs), such as spermidine (SPD) and spermine (SPM), are considered significant plant growth regulators (PGRs) that play a role in controlling plant growth and senescence, including enhancing resistance to heat stress [46,47]. The composition of polyamines in microalgae varies in proportions, and changes in polyamine content in response to changes in the environment. It was found that spermine was the main polyamine altered in diatoms under silicate stress [33], whereas in the present study, putrescine and spermine were the main polyamines altered in response to the change of temperature conditions. A similar situation was observed in different salt stresses. Under high-salinity stress, *Skeletonema costatum* significantly increases its polyamine levels, particularly the content of free polyamines, whereas *Prorocentrum donghaiense* primarily regulates its bound spermidine and free spermine. Under low-salinity stress, *Skeletonema costatum* enhances the production of free putrescine, while *Prorocentrum donghaiense* exhibits an overall increase in the levels of all forms of polyamines [48]. According to our results, the main polyamine controlling the growth rate in *Skeletonema dohrnii* under temperature change was spermidine. The beneficial, general stimulatory effect of putrescine in plants has long been known. However, the specific role of putrescine in microalgae is not clearly understood. It has been found that a low temperature increases the putrescine content of maize [49], and a similar situation occurred in the low-temperature group in the present study. Changes in putrescine concentration appear to act on genes at the transcriptional level. For example, putrescine alters the level of heat-shock protein gene expression [50]. According to previous studies, spermine treatment also increased the expression levels of stress-related genes that protect seedlings from stress damage [51]. In our study, we found that the content of spermine increased at low temperatures compared with normal temperatures, suggesting that spermine is involved in stress alleviation during low-temperature stress. Interestingly, we did not find an increase in spermine content in the high-temperature group, which suggests that spermine is not involved in stress relief during high-temperature stress. Polyamines can regulate the up- or down-regulation of gene expression either directly or by stimulating the phosphorylation of regulatory proteins, such as transcription factors. For example, a protein kinase in rice is regulated by spermidine at the transcriptional and translational levels [52]. Another example of the covalent binding of polyamines to proteins is observed in the biosynthesis of hypusine. In this process, the butylamino group of spermidine is utilized to facilitate the post-translational modification of the precursor of the eukaryotic translation initiation factor 5A (eIF5A), converting lysine into hypusine [53]. Hypusine-modified eIF5A is essential for the growth of all eukaryotic cells. This could potentially explain the higher growth rates observed under high-temperature conditions. Additionally, the content of spermidine may play a role in regulating diatom cell division.

### 3.3. Response of Polyamine Oxidase Gene Expression to Temperature

The metabolism of polyamines is very closely related to two enzymes. One is polyamine synthase, and the other is polyamine oxidase. Polyamines are involved in the process of linking stresses in algae facing environmental stresses. Based on the results of the study, we found that the expression of polyamine oxidase was down-regulated in both the high-temperature and low-temperature groups relative to the normal-temperature group (Figure 6). Previous studies have also found that diatom polyamine oxidase genes are down-regulated in response to silicate stress [33]. This suggests that the algal cells need a higher concentration of polyamine to maintain the physiological activity of the cells. Elevated PA levels were found to make plants stress-tolerant in the study [54]. It was found that AtPAO2 and AtPAO5 expression was up-regulated under saline stress; however, AtPAO1, AtPAO3, and AtPAO4 were not affected by salinity treatment [55]. Sagor et al. [56] generated a series of *A*. *thaliana* mutant lines in which the PAO gene was knocked out. The AtPAO1 and AtPAO 5 double mutants were tolerant to saline and dehydration stresses, while the double mutant AtPAO 2–AtPAO 4 was sensitive to saline and dehydration stresses, compared to the wild type (WT). It was further observed that PAO activity was reduced to 62% and Na^+^ uptake was reduced to 75% in the former double mutant compared with the wild type. In addition, the level of spermine was higher in the mutant than in the wild type. Tolerance of the mutant phenotype may be related to the down-regulation of PAO metabolism leading to increased levels of polyamines. Our study revealed a decrease in PAO gene expression in the low- and high-temperature groups compared to the normal-temperature group, which tentatively suggests that polyamines are involved in the mitigation of temperature stress in diatoms.

## 4. Materials and Methods

### 4.1. Source and Cultivation of Algal Species

The experimental species *Skeletonema dohrnii* was collected from the Yellow Sea and preserved in f/2 culture medium. The algae were kept at 25 °C, with a light exposure of 100–120 µmol photons m^−2^·s^−1^, and a light exposure to darkness ratio of 12 h:12 h. The experimental algae were cultivated in artificial seawater (ASW) under the same storage conditions as the original algal species. The specific composition of ASW is shown in Table 1; final salinity is 35 PSU.

### 4.2. Experimental Training Conditions

The experiment involved three temperatures: 15 °C, 25 °C, and 28 °C. These concentrations corresponded to low (15 °C), normal (25 °C), and high (28 °C) temperatures. Each concentration was mixed with 300 mL of culture medium in 3 replicates. The algal cells were cultured semi-continuously in a 500 mL polycarbonate bottle and diluted every 3 days to facilitate cell transfer during the exponential growth phase.

### 4.3. Determination of Growth Rate

We counted under a light microscope using a blood cell counting plate and then calculated the growth rate (d^−1^) using Equation (1).

(1) $$\mu =\left(\mathrm{Ln}N_{1}-\mathrm{Ln}N_{0}\right)/t$$

where *N*_0_ is the cell density at the beginning of the algae dilution cycle, *N*_1_ is the cell density at the end of the algae dilution cycle, and *t* is the duration of the algae dilution cycle.

### 4.4. Determination of Fv/Fm

A hand-held algal fluorometer (cuvette type) (model: AquaPen AP-C100; PSI., Ltd., Prague, Czech Republic) with an optical glass cuvette and FluorPen system software (version number 1.1) was utilized to measure chlorophyll fluorescence parameters. The maximum photochemical quantum yield of PSII, represented by the chlorophyll fluorescence parameter *F_v_/F_m_*, is typically determined using Equation (2):

(2) $$\frac{F_{v}}{F_{m}}=(F_{m}-F_{0})/F_{m}$$

where *F*_0_ and *F_m_* are the minimum and maximum fluorescences measured when the test algal sample is dark-adapted, respectively [57]. Dark adaptation time is 10 min.

### 4.5. Determination of Chlorophyll Concentration

We introduced 5 mL of algal solution into a GF membrane, then transferred the membrane to a brown vial. After adding 5 mL of 90% acetone to the vial, we allowed the extraction process to continue at −20 °C for 24 h. Subsequently, we utilized a TriLog fluorescence analyzer (Turner, CA, USA) to determine the chlorophyll *a* content and compute the Chl-a concentration.

### 4.6. Determination of Biogenic Silicon Concentration

A 5 mL sample of algal solution was passed through a 0.6 µm PC membrane for filtration. The membrane, now containing algae, was transferred to a centrifuge tube filled with 10 mL of ultrapure water. The tube was agitated to ensure complete dispersion of algae from the membrane into the water. This mixture was then subjected to heat treatment in a 100 °C water bath for 10 min. Following this, the heated solution underwent centrifugation and filtration. The resulting filtrate and residue were subsequently analyzed to determine biosilica content, following the methodology outlined by Strickland and Parsons [58].

### 4.7. Determination of Polyamine Content

Using high-performance liquid chromatography (HPLC), polyamines were quantified in accordance with Aizi. Shanghai Fuda Detection Technology Group Co., Ltd. (Tianjin, China) conducted the analysis to measure the concentration of polyamines in this research.

### 4.8. Design of Primer Sequences

Based on unpublished transcriptome data from the literature [33], primer 5.0 was used to design primers for polyamine oxidase genes and housekeeping genes (Table 2). The CaM gene is a housekeeping gene, and CaM is a calmodulin protein that is widespread in eukaryotic cells and is highly evolutionarily conserved. Calmodulin itself has no enzymatic activity and is biologically inactive in the absence of Ca^2+^.

### 4.9. Reverse Transcription

A reagent kit from Takara was used for this operation. First, 1 μL Oligo dT Primer, 1 μL dNTP Mixture, 4 μL RNase Free dH_2_O, and 4 μL RNA from diatoms were added to the PCR tube for mixing, and then the PCR tube containing the solution was heated in a water bath at 65 °C for 5 min; the PCR tube was placed on ice after the heating was completed, and 4 μL of 5× Primer Script II Buffer, 0.5 μL of RNase Inhibitor, 1 μL of Primer Script II, and 4.5 μL of RNase Free dH_2_O were added to the tube for mixing. Then, the PCR tube containing the mixture was put into the PCR tube containing the mixture, put into the Eppendorf PCR instrument and run according to the set reaction program, and cDNA was obtained. The reaction program was as follows: 42 °C for 60 min, 95 °C for 5 min, 72 °C for 15 min, and storage at 4 °C.

### 4.10. Real Time Fluorescence Quantitative PCR

After conducting primer suitability PCR screening, it was determined that the optimal annealing temperature (Tm) for the polyamine oxidase gene and CaM gene primers was 60 °C, within a temperature gradient range of 53–60 °C. CaM was used as an internal reference gene for quantitative experiments using cDNA from *Skeletonema dohrnii* vulgaris cultured at various silicate concentrations, following a two-step method. Three parallel experiments were conducted, with negative controls included to minimize systematic errors from sampling and instruments. The mRNA levels of polyamine oxidase genes were measured using the Step One Plus real-time PCR system under culture conditions with different silicate concentrations.

### 4.11. Data Analysis

The physiological data of algal cells in this study were analyzed using GraphPad Prism 9.4 for statistical evaluation. A t-test was used as a post-test for one-way ANOVA with a significance level of *p* < 0.05, indicating a statistical difference in the data.

Following the completion of real-time fluorescence quantitative PCR, the data were analyzed using the ABI software (version number 2.3, Applied Biosystems, USA). The relative expression level of the polyamine oxidase gene mRNA under various environmental conditions was calculated using the 2^−∆∆ Ct^ method based on the Ct values of the target gene and the CaM gene. A temperature of 25 °C was used as the control for gene expression levels.

## 5. Conclusions

In this study, we investigated the physiological response and molecular mechanism of *Skeletonema dohrnii* in diatoms to different temperature stresses. We focused on the growth rate, biogenic silica content, polyamine level and polyamine oxidase expression of *Skeletonema dohrnii* in response to temperature. It was shown that the growth rate of *Skeletonema dohrnii* was significantly enhanced by increasing temperature. The low temperature contributed to the increase in biosilicon content. The polyamine composition of *Skeletonema dohrnii* was different between low- and high-temperature stresses. Both low and high temperatures led to a decrease in the gene expression level of PAO. These results may provide data for the study of the molecular regulation mechanism of diatoms facing abiotic stress.

## Figures and Tables

**Table 1:** Composition of artificial seawater.

Composition	Final Concentration
NaCl	4.20 × 10^−1^ M
Na_2_SO_4_	2.88 × 10^−2^ M
KCl	9.39 × 10^−3^ M
NaHCO_3_	2.38 × 10^−3^ M
KBr	8.40 × 10^−4^ M
H_3_BO_3_	4.85 × 10^−5^ M
NaF	7.15 × 10^−5^ M
MgCl_2_·6H_2_O	5.46 × 10^−2^ M
CaCl_2_·2H_2_O	1.05 × 10^−2^ M
SrCl_2_·6H_2_O	6.38 × 10^−5^ M

**Table 2:** Primer sequences.

Primer Name	Sequence
PAO-U	GCGGCTGAGAGAGCAGCAAACTCCC
PAO-D	CAATAACACGTGGAGGACGTCG
CaM-U	TCGATGTTGATGGTGGTGGAA
CaM-D	TCGGGGTCGTCTTTGCTGTT

## Data Availability

Data are contained within the article.
